# Employing Kirkpatrick’s framework to evaluate nurse training: an integrative review*

**DOI:** 10.1590/1518-8345.7250.4431

**Published:** 2025-02-03

**Authors:** Fernanda Maria de Miranda, Bruna Vasconcelos dos Santos, Vicki Leigh Kristman, Vivian Aline Mininel

**Affiliations:** 1 Universidade Federal de São Carlos, São Carlos, SP, Brazil; 2 Scholarship holder at the Coordenação de Aperfeiçoamento de Pessoal de Nível Superior (CAPES), Brazil; 3 Lakehead University, Department of Health Sciences, Thunder Bay, ON, Canada; 4 Universidade Federal de São Carlos, Departamento de Enfermagem, São Carlos, SP, Brazil

**Keywords:** Continuing Education, Nurses, Evaluation of the Efficacy-Effectiveness of Interventions, Review, Methods, Inservice Training

## Abstract

to evaluate the evidence on the use of Donald Kirkpatrick’s framework in nursing training evaluation.

integrative literature review in the Latin American and Caribbean Health Sciences Literature, Medical Literature Analysis and Retrieval System and Web of Science databases. Studies that answered the review question “Which is the evidence in using Donald Kirkpatrick’s framework to evaluate training in the nursing workplace?” published in Portuguese, English, or Spanish were included.

out of 108 studies retrieved, thirteen were included. The majority evaluated the four levels proposed in the model (reaction, learning, behavior, and results) or, at least, a combination of the first three ones. Different instruments were used to evaluate nursing training, mainly in quantitative approaches for reaction and learning levels and qualitative for behavior and results levels. This approach highlights the flexibility of the model and the importance of choosing a reliable set of instruments, which is crucial to qualify the analysis at each level.

Kirkpatrick’s model has been used worldwide to evaluate training in the nursing field and has been shown to be suitable for it, as long as there is an appropriate selection of instruments at each level.

## Highlights

**(1)** Kirkpatrick’s framework is effective for evaluating various nursing training.

**(2)** The framework upholds the choice of measuring instruments for each level.

**(3)** The four-levels or a combination of the first three were the most commonly used to evaluate training.

**(4)** The four-levels or a combination of the first three were the most common to evaluate training.

**(5)** Evaluation of results in organizational practices is the most challenging level.

## Introduction

Training healthcare personnel has been incorporated as a strategy for talent retention in human resources management. Training and development practices at the workplace are described as dynamic and continuous processes aimed at promoting social advances, increasing resolution and fostering better health outcomes^(1)^.

Training can be an opportunity for professional development and increased productivity, creativity, and innovation. These are the main aspects of high-quality services and teams effectiveness^(2)^. Lack of training can negatively influence perception of stress, increase turnover and stress, and reduce work performance^(3)^.

Training strategies are key to improve health systems in low-income countries^(4)^. The development of workers through training is a critical indicator of effective management^(5)^. In Brazil, training is also political, since knowledge interacts as an essential part of the power struggle and meaningful learning could lead to deep-rooted changes in healthcare^(6)^.

Evaluating training programs can help managers to understand the effectiveness and sustainability of the learning process to produce changes in the short or long term. However, selecting a suitable framework for evaluation is not an easy task and many interventions struggle with low or very low levels of evidence for targeted outcomes^(4)^.

In 1959, Donald Kirkpatrick published a framework to support managers to evaluate the results of training and development practices among workers and organizational systems^(7-8)^, based on summative assessments. The model is composed of four levels of evaluation: Reaction, Learning, Behavior and Results^(7)^. Reaction (level I) encompasses the participant’s perceptions of their learning experience, program structure, content, teaching methods, and instructional aspects, such as materials and the quality of instruction. Learning (level II) is related to changes in attitudes, perceptions, knowledge and skills. Behavior (level III) evaluates the knowledge transfer to the workplace through behavioral changes. Finally, the Results level (level IV) evaluates the applicability of intervention results to change the organizational practice and improve the outcomes^(7-8)^.

Managers and researchers should carefully consider adopting an evaluation system based on this framework, as the implementation of isolated levels is not recommended and higher level of evaluation do not lead to more valuable results if they are isolated^(7)^.

The Kirkpatrick model has been widely accepted in the scientific community to support development and training in several areas, including health^(9-14)^. Several improvements were described by the Kirkpatrick Group since the first publication, arriving at a new model called the New World Kirkpatrick model in 2016^(15)^. Despite the improvements, there are criticisms regarding the applicability of the Kirkpatrick model^(16-18)^, the major concern being the difficulty in implementing all four levels^(16)^. These criticisms prompted this review to ensure the method’s applicability in nursing. The aim of this study is to evaluate the evidence on the use of Donald Kirkpatrick’s framework in the evaluation of nursing training.

## Methods

### Type of study

We used an integrative literature review, based on the Whittemore and Knafl framework, carried out in five stages: problem identification; literature search/screening; data evaluation; data analysis; presentation of the review^(19)^. We choose this methodological structure to ensure a systematic approach to evidence synthesis. The intended outcome is to obtain a comprehensive understanding of the use of Kirkpatrick’s framework in nursing and the identification of gaps for future studies^(19)^.

This integrative review was registered on the Open Science Framework (OSF) platform on May 20^th^, 2022, and the protocol can be accessed at osf.io/uprv7, with the following DOI 10.17605/OSF.IO/JQ8U9^(20)^.

### Locus

This integrative review was conducted in the municipality of Thunder Bay, located in the province of Ontario (ON), Canada.

### Period

The study period was September 2022 to November 2023.

### Population

We used the review question: “Which is the evidence in the use of the Donald Kirkpatrick framework to evaluate nursing workplace training?” was used. The formulation of the research question was developed from the PCC strategy namely: Population (P): nursing; Concept (C): Donald Kirkpatrick’s four-level framework; Context (C): evaluation of workplace training.

### Selection criteria

Studies were included if they addressed the research question and if they were published in English, Spanish, or Portuguese between 2006 to 2023, with full content available. The time frame was based on the year of publication of the third edition of the book “Evaluating training programs” where some interpretations of the traditional framework were updated^(7)^. Studies that did not meet the inclusion criteria or did not clearly describe the instruments used were excluded. We also removed duplicates, literature reviews, and gray literature.

### Sample definition

The search was performed in November 2022 from Thunder Bay, ON, Canada by FMM, end updated in August 2023. The Latin American and Caribbean Health Sciences Literature (LILACS), Medline (via PubMed), and Web of Science (WoS) bibliographic research databases were used.

Search strategies (strings) were based on the Health Sciences Descriptors (DeCS) and the Medical Subject Heading (MeSH). After analysis of exploratory searches, we decided to include the keyword “Kirkpatrick’’ to reduce the scope of the search. The final strategy used was: (”Education, continuing” OR “Continuous Learning” OR “Learning, Continuous” OR “Lifelong Learning” OR “Learning, Lifelong” OR “Life-Long Learning” OR “Learning, Life-Long” OR “Learnings, Life-Long” OR ’Life Long Learning” OR “Life-Long Learnings” OR “Continuing Education”) AND ((“nurses” OR ’Nurse” OR “Personnel, Nursing” OR “Nursing Personnel” OR “Registered Nurses” OR ’Nurse, Registered” OR “Nurses, Registered” OR “Registered Nurse”) OR (“nursing, team”)) AND (“Kirkpatrick”) for MEDLINE and WoS. For LILACS, it was decided to use only the keyword “Kirkpatrick” considering the theme specificity and the quantity of studies retrieved in the original string.

After searching the databases, the files generated in the BIBTXT or RIS format were exported to the Rayyan^®^ review manager^(21)^ which supported the initial screening of abstracts and titles using a semi-automation process. The selection of articles was carried out in two blind stages by two different researchers (FMM and BVS); the first screening was completed by reading the titles and abstracts of the retrieved documents; the second screening involved a complete review. Conflicts were discussed until a consensus was reached. A third researcher (VAM) was available to decide any persistent conflict.

### Data collection

The data was extracted using an analytical matrix built in Microsoft Excel^®^ containing information of the characteristics of the studies (authors, year of publication, country of publication, objective, type of study, sample and results) and information on the use of the Kirkpatrick model (levels used, elements, timing, evaluation criteria and measures).

### Data treatment and analysis

We analyzed the data through reduction and comparison to identify patterns, themes, or relationships^(19)^. Subsequently, a descriptive analysis based on the theoretical-conceptual framework adopted was carried out, considering the precepts of Donald Kirkpatrick’s framework^(7)^.

This review followed the guidelines of the Preferred Reporting Items for Systematic Reviews and Meta-Analyses (PRISMA) for reporting systematic reviews^(22)^. PRISMA was designed for systematic reviews of studies evaluating the effects of health interventions; however, the items on the checklist are applicable to reports of systematic reviews in other areas or evaluating other types of interventions^(22)^. Thus, all PRISMA section were used to guide the review report, except for topics not applicable to this review’s approach, including analyses of the risk of bias, determination of measures of effect, and analyses of possible causes of heterogeneity. The effectiveness of the Kirkpatrick framework through the training effectiveness results shown in each article included in the sample selected for this study was analyzed. To do this, both the training design and the use of Kirpatrick levels were considered, the configuration of the instrument used, the timing of the evaluation and other aspects.

## Results

**Figure 1 -:**
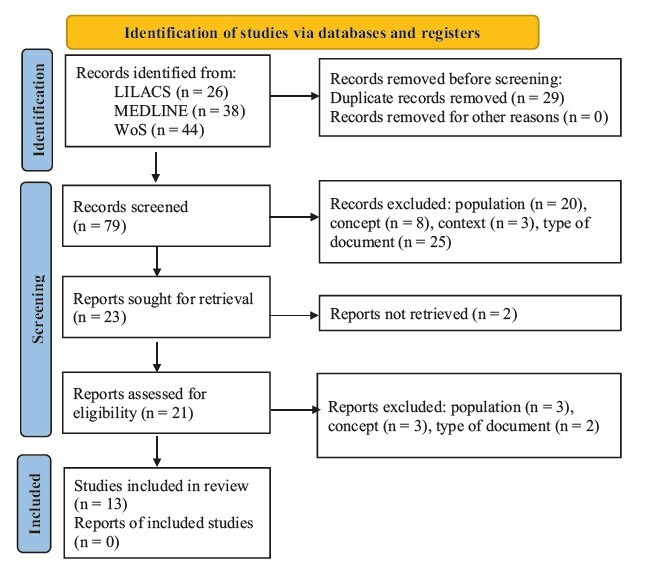
PRISMA^(22)^ workflow for sample selection. Thunder Bay, ON, Canada, 2023

One hundred and eight articles were retrieved and 13 were included in the final sample (Figure 1).

**Figure 2 -:**
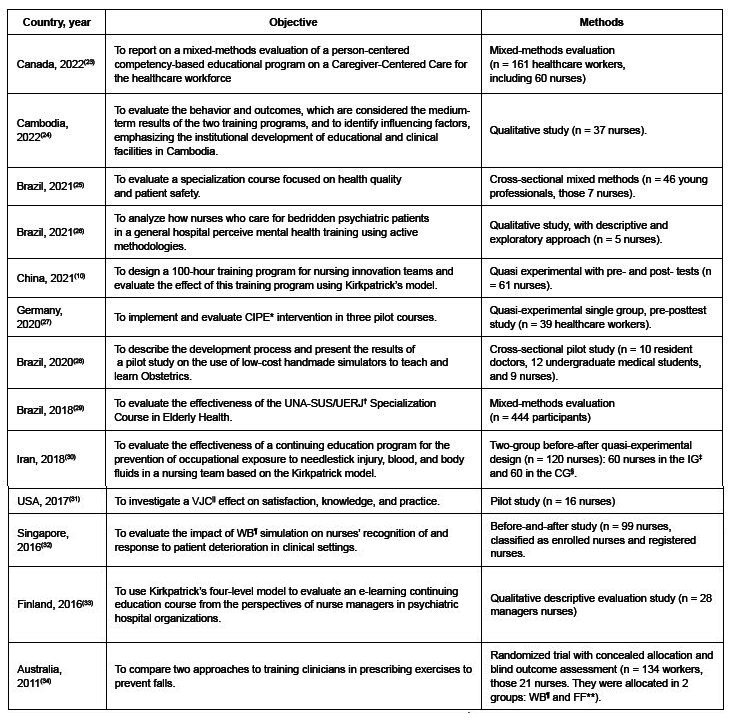
Sample characterization (n = 13). Thunder Bay, ON, Canada, 2023

Figure 2 shows the 13 articles characterized by country, year of publication, objective and methods.

*CIPE = Continuing interprofessional education; ^†^UNA-SUS/UERJ = *Universidade Aberta do Sistema Único de Saúde/Universidade Estadual do Rio de Janeiro*; ^‡^IG = Intervention group; ^§^CG = Control group; ^||^VJC = Virtual journal club’s; ^¶^WB = Web-based; **FF = Face-to-face

**Figure 3 -:**
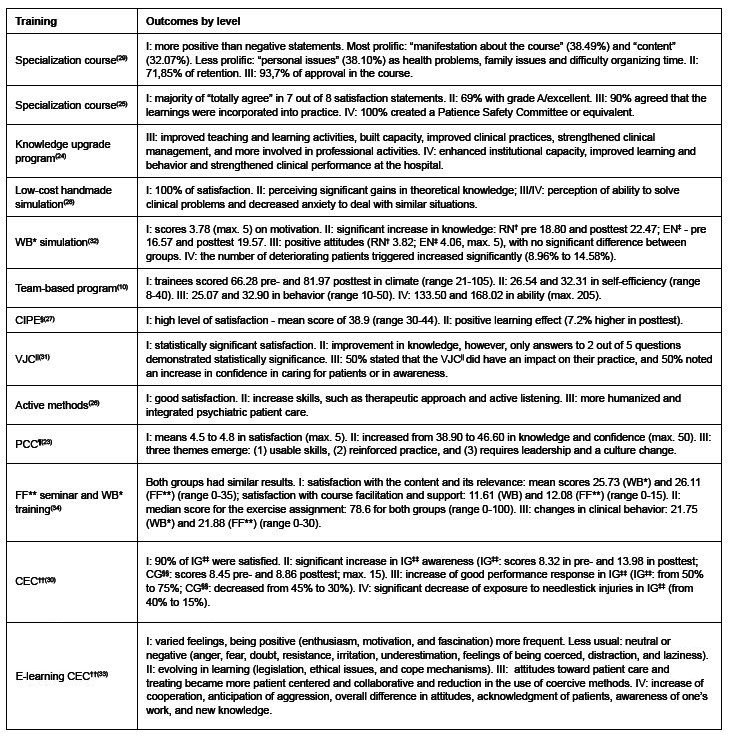
Using the four-levels Kirkpatrick model to evaluate training results (n = 13). Thunder Bay, ON, Canada, 2023

The Kirkpatrick model has been useful in training, continuing education and updating courses, as well as in specialization programs with individual-centered and team-based interventions. Figure 3 shows the use of the four-levels Kirkpatrick model to evaluate training results:

*WB = Web-based; ^†^RN = Registered nurse; ^‡^EN = Enrolled nurse; ^§^CIPE =Continuing interprofessional education; ^||^VJC = Virtual journal club’s; ^¶^PCC = Person-centered and competency based program **FF = Face-to-face; ^††^CEC = Continuing education course; ^‡‡^IG = Intervention group; ^§§^CG = Control group

Kirkpatrick’s framework was effective in evaluating all the training identified, considering the measurable and prolific outcomes of the samples by level in different types of training. Consistency in compliance with the guidelines was observed, demonstrating their versatility and flexibility.

Brazil was the only country with more than one publication^(25-26,28-29)^. Quasi-experimental approaches^(10,27-28,30-32)^ followed by qualitative research^(24,26,33)^, mixed-methods research^(23,25,29)^ and one experimental study^(34)^ were found.

Most of the studies used all four levels of Kirkpatrick’s Framework^(10,25,28,30,32-33)^ or a combination of the first three levels^(23,26,29,31,34)^. One article evaluated the intervention with a combination of levels I and II^(27)^ and another with a combination of levels III and IV^(24)^.

**Figure 4 -:**
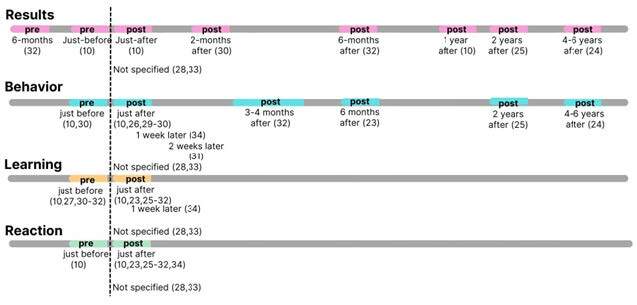
Multiple data points were collected before and after the intervention using the four levels of Kirkpatrick’s model (n = 13). Thunder Bay, ON, Canada, 2023

Figure 4 shows a timeline for the implementation of these levels.

Two studies^(28,33)^ did not identify the timeline for data collection. They only described that there was cross-sectional data collection considering the availability of the intervention for a period of time such as “since 2014”^(28)^ or “between 2008 and 2011”^(33)^. None of the studies had explicit follow-up designs, although some of them collected data long after the intervention^(10,24-25,32)^. In one of them, the authors cited a previous data collection after the intervention, but did not present or compare the data, nor did they characterize a follow-up study^(24)^.

Different instruments have been used to evaluate interventions, based on the specificity of the investigation; however, a main approach or a standard instrument for evaluating any level of the Kirkpatrick Framework was not found. Considering the relevance of measurement tools for the four levels of Kirkpatrick’s model and the lack of consensus on the best way to do it (and this is the richness of the model – it can be customized according to with study), it was decided to synthesize the instruments that were used in each selected study to help future nurses in this topic.

Quantitative instruments were the most common. Table 1 shows the quantitative instruments used to evaluate the training programs, their measures (items and range), and whether the study described some source of reliability using Cronbach’s α.

**Table 1 -:** Quantitative instruments applied to evaluate the four-level Kirkpatrick’s framework (n = 13). Thunder Bay, ON, Canada, 2023

**Level: Instrument Description**	**Item**	**Range**	α*
I: Five-point Likert scale for learner’s satisfaction^(23)^	5	1-5	n/s^†^
I: Five-point Likert scale’s questionnaire: “material and syllabus”, “assignments”, “tutoring”, and “learning support structure”^(25)^	4	1-5	n/s^†^
I: Reaction assessing tool including exogen aspects, (e.g., internet access) and endogenous aspects (e.g., material) for each module and the entire course^(29)^	9	1-4	n/s^†^
I: NOIC^‡^ with 5-point Likert scale^(10)^	21	1-5	0.94
I: ABC-SAT^§^, domains: a = affective, b = behavioral, c = cognitive^(27)^	a = 3	0-12	0.60
	b = 2	0-8	0.66
	c = 6	0-24	0.83
I: Five-point Likert scale statements related to satisfaction, potentiality for optimal learning and bad feelings’ prevention^(28)^	3	1-5	n/s^†^
I: Five-point Likert’s scale questionnaire, domains: c = content, t = teachers, f = facilities^(30)^	c = 5	1-5	0.87
	t = 4		
	f = 3		
I: Five-point Likert scale’s survey related to usability, format, discussion opportunities, participation and time adequacy^(31)^	6	1-5	n/s^†^
I: IMMS^||(32)^	4	1-5	0.79
I: Self-reported satisfaction questionnaire, domains c = “content and its relevance” and s = “support and facilitation”^(34)^	c = 35	0-35	0.92
	s = 15	0-15	0.77
II: 10-questions education knowledge and confidence test^(23)^	10	5-50	0.92
II: Four-Processual tests (one for each learning module)^(25)^	n/s^†^	0-10	n/s^†^
II: The innovation self-efficacy questionnaire^(10)^	8	1-5	0.86
II: Self-developed written knowledge test regarding key topics of the intervention, each with 4 possible answers^(27)^	9	0-18	n/s^†^
II: Five-point Likert scale’s statements: “(...) was used to increase my theoretical knowledge” and “(...) was used to increase my ability to solve clinical problems”^(28)^	2	1-5	n/s^†^
II: Awareness questionnaire^(30)^	15	0-15	0.87
II: 5-question closed-end test (2 had more than one correct answer)^(31)^	5	0-5	n/s^†^
II: Metacognition Questionnaire^(32)^	30	n/s^†^	n/s^†^
II: 1-hour knowledge test^(34)^	30	0-30	n/s^†^
II: Assignment submission requiring a description of an exercise program tailored to a hypothetical client scenario^(34)^	1	0-100	n/s^†^
III: Self-reported questionnaire about behavior regarding patient safety protocols^(25)^	9	1-5	n/s^†^
III: NIBS^¶(10)^	10	1-5	0.90
III: Two closed-ended statements about decreased of anxiety/stress and ability of solve a clinical problem/situation^(28)^	2	1-5	n/s^†^
III: The performance questionnaire^(30)^	15	0-15	0.78
III: Self-reported Knowledge transfer at workplace questionnaire^(32)^	14	1-5	0.94
III: Self-reported satisfaction questionnaire, domain b = “change in clinical behavior”^(34)^	b = 30	0-30	0.84
IV: Questionnaire of agreement about the implantation of five goals for patient safety workplace after the course^(25)^	6	1-5	n/s^†^
IV: Scale of Clinical Nursing Staff Innovation Ability^(10)^	41	0-205	0.82
IV: The questionnaire for exposure to sharp objects, blood and body fluids^(30)^	53	n/s^†^	0.89

*α = Cronbach’s α; ^†^n/s = Not specified; ^‡^NOIC = The Nurse Organizational Innovation Climate Scale; ^§^ABC-SAT = The ‘Affective-Behavioral-Cognitive-Satisfaction questionnaire’; ^||^IMMS = The Instructional Materials Motivation Survey; ^¶^NIBS = Nurse Innovation Behaviour Scale

A great variability of instruments regarding quantitative approaches was found. Level I was mostly evaluated by a five-point Likert scale for items such as satisfaction^(23,25,27-28,30,32,34)^, immersion^(28,31)^, climate^(10)^, relevance and confidence^(32)^. Both knowledge and awareness approaches were used on level II. The most common was closed-end learning tests^(25,27,29-31,34)^, followed by self-rated questionnaires^(10,23,32)^. Level III and IV quantitative measures included trainees’ perception of knowledge transfer to the workplace through closed-end questionnaires^(10,25,28,30,32-34)^ or scales^(10)^.

Although less frequent than quantitative, the use of a qualitative approach was found in behavior level^(23-24,26,29,31,33)^ followed by reaction^(26,29,33)^, results^(24,28,33)^ and learning^(26,33)^. Seven^(23-24,26,28-29,31,33)^ studies used qualitative instruments to evaluate training programs, however only two^(24,26)^ of them did so without a quantitative piece. Semi-structured interviews^(23-24,26)^, questionnaires with open questions^(28,33)^, descriptive analysis of the key words in students’ projects^(29)^, qualitative research^(31)^, qualitative analysis based on the participants’ narratives^(29)^, and adherence after the first month of the intervention^(29)^ were identified. The most prevalent type of qualitative analysis was thematic analysis^(24,26,28-29)^. Most of the studies did not describe reliability or validity of the qualitative instruments, with the exception of Koto-Shimada^(24)^ who carried out a pilot test. Other work-related measures have been also reported, i.e., clinical records and patient information^(32)^, self-reported exposure to needlestick injury questionnaire^(30)^.

Choosing a measurement tool is crucial to evaluating the effectiveness of any intervention, especially when it comes to training. This step is embodied into Kirkpatrick’s model and should be carefully selected by nurses in order to qualify the analysis of each level. The more precise the tool (i.e., more in line with the object of study), the greater the chances of a reliable evaluation.

## Discussion

This review has shown that the Kirkpatrick Framework has been widely used in the evaluation of nurses’ training and also in other areas of health^(11)^. Our sample depicted in this study indicated consistency in compliance with the guidelines and the main training outcomes indicate that this model is useful to evaluate effectiveness, with positive results at each level in different types of training interventions.

The evaluation process converged with the orientation of the framework which focuses on participant outcomes^(7)^. However, one study approached the participants’ managers and a complementary view emerged, as exemplified in the quote: “the nurses did not experience the change in the atmosphere and attitudes in the same way as the nurse managers had observed”^(33)^. The inclusion of the leadership perspective can be positive, especially to obtain feedback on knowledge transfer to the workplace (level III) or changes in practice or organizational outcomes (level IV) considering their supervisory role.

The most common type of baseline information was on Learning level^(10,23,27,30,32)^. The Behavior and Results levels were the most divergent levels regarding timing. There are no guidelines in Kirkpatrick’s Framework on the best time to evaluate each level^(7,15)^ and we cannot support a more common or assertive time for data collection, considering that none of the studies converge on the best time. However, it is important to point out that training programs expect short-, medium- and long-term outcomes. Therefore, selecting a specific time (e.g., before, during, soon after, longer after) or period (e.g., follow-ups) is essential for a comprehensive evaluation that considers that feeling of reaction will fade over time and the most important outcomes will appear over time^(8)^.

The Kirkpatrick model is a flexible and adjustable framework that embeds different tools for evaluating training outcomes (Reaction, Learning, Behavior and Results), which reinforces its power, since the realities, training and contexts of each organization are completely different. However, precisely because it does not indicate a specific methodological standard, authors must be careful to ensure the validity and reliability of the information collected^(17)^.

Both the number of studies and the diversity of instruments decreased as the level of evaluation increased, it has been observed. The literature review indicates that the Results’ level is the most difficult to achieve. There is acknowledged that level IV “could identify the added value to society of a given educational program, as it makes it possible to evaluate not only of the application of intervention projects in the practice of health professionals, but also the results of their use in a local context”^(29)^. However, half of the samples in this review did not report measuring this level. The most common reason reported for the exclusion of level IV was the need for medium- and long-term follow-up periods^(26)^ and the complexity of relating the outcomes with the training program, distinguishing them from many other factors that could affect the results^(31)^. Results evaluation (level IV) is the most challenging because it must consider the transformation or impacts of training on organizational practice, which requires other types of instruments, organizational information and the point of view of other stakeholders, as well as more time. In this sense, similar data was found regarding the perceived relevance of levels III and IV and the difficulty to implement these levels^(17)^.

There is some criticism about the rigidity of this framework, which can lead to essential aspects of the evaluation, such as the formative vision being missed^(16,18)^. Only one study reported data collection in each module and at the end of the course at the reactions level^(29)^. No other experience was identified at the follow-up. This evidence shows that, despite the diversity in the perspective of outcomes evaluation tools, especially with the use of qualitative approaches to capture the trainees’ perspective, all the experiences reported were in a summative or transversal view.

Trainers are not using only the highest levels, which is a concern of the criticism of Kirkpatrick’s model^(16-18)^ it has been observed. Six articles managed to reach Level four and another five used a combination of the first three levels; accounting for 85% of the sample. This indicates that nursing trainers are attempting to use all four levels of the model to collect information on outcomes, despite the challenge regarding the Results level. This reaffirms the researchers’ commitment to the framework’s recommendation to use a complete model for greater accuracy^(7-8)^.

The relevance of this review is the fact that it synthesizes the recognition of the operationalization of the framework. A large bibliometric analysis found the Kirkpatrick model to be a trending topic of interest after the 2000s, including 20.7% of its sample as some kind of literature review publications. However, most studies aimed to criticize the model or identify its benefits, and little review evidence was found on the types and characterization of evaluation tools and their application in a specific field, such as nursing^(11)^.

This study has methodological limitations. The multiple research designs of the publications included made it impossible to produce evidence through meta-analysis or meta-synthesis techniques. Considering the nature of the integrative review, the aim is not to critically appraise the quality of the studies and therefore, the quality of the study was not used an inclusion criterion.

We encourage further research to identify the best constructs for evaluating each level, to expand the implementation of level IV and to foster scientific conversation about the methodological orientation for composing the chain of evidence between the levels, as proposed by the New World Kirkpatrick model^(15)^. Moreover, our literature review identified limited longitudinal studies with consecutive follow-ups for data collection, which is highly recommended for achieving high-level evidence regarding causal pathways.

## Conclusion

Kirkpatrick’s four-level Framework is a suitable choice in terms of adapting evaluation instruments to the training design. The selection of a reliable set of instruments is crucial to qualify the analysis of each level, with Likert scales being the most common choice for trainers. This model is prolific for evaluating training in the nursing field but requires a careful choice of instruments for each level. Many qualitative and quantitative measurement tools have been identified that can be useful for practitioners and academics in further evaluation and research.
