# Supplementary material for: Employing Kirkpatrick’s framework to evaluate nurse training: an integrative review
Source: Rev Lat Am Enfermagem. 2025 Feb 3;33:e4431. doi: 10.1590/1518-8345.7250.4431 (PMC11789729; doi:10.1590/1518-8345.7250.4431)
Supplement: Translation

# Translation: Uso de la metodología de Kirkpatrick para evaluar la formación de los profesionales de enfermería: una revisión integradora*

**DOI:** 10.1590/1518-8345.7250.4430

**Authors:** Fernanda Maria de Miranda, Bruna Vasconcelos dos Santos, Vicki Leigh Kristman, Vivian Aline Mininel

**Affiliations:** 1 Universidade Federal de São Carlos, São Carlos, SP, Brasil; 2 Becaria de la Coordenação de Aperfeiçoamento de Pessoal de Nível Superior (CAPES), Brasil; 3 Lakehead University, Department of Health Sciences, Thunder Bay, ON, Canada; 4 Universidade Federal de São Carlos, Departamento de Enfermagem, São Carlos, SP, Brasil

**Keywords:** Educación Continua, Enfermeras y Enfermeros, Evaluación de Eficacia-Efectividad de Intervenciones, Revisión, Métodos, Capacitación en Servicio

## Abstract

evaluar la evidencia sobre el uso del modelo de Donald Kirkpatrick en la evaluación de la formación en enfermería

revisión bibliográfica integradora en las bases de datos Literatura Latinoamericana y del Caribe en Ciencias de la Salud, *Medical Literature Analysis and Retrieval System* y *Web of Science*. Se incluyeron los estudios que respondían a la pregunta de revisión “¿Cuál es la evidencia para el uso del modelo de Donald Kirkpatrick para evaluar la formación en el trabajo de enfermería?” publicados en portugués, inglés o español.

de los 108 estudios recuperados, se incluyeron trece. La mayoría evaluaba los cuatro niveles propuestos en el modelo (reacción, aprendizaje, comportamiento y resultados) o al menos una combinación de los tres primeros. Se utilizaron diferentes instrumentos para evaluar la formación en enfermería, principalmente enfoques cuantitativos para los niveles de reacción y aprendizaje y enfoques cualitativos para los niveles de comportamiento y resultados. Este enfoque pone de manifiesto la flexibilidad del modelo y la importancia de elegir un conjunto fiable de instrumentos, lo cual es fundamental para cualificar el análisis en cada nivel.

el modelo de Kirkpatrick se ha utilizado en todo el mundo para evaluar la formación en enfermería y ha demostrado ser adecuado para este fin, siempre que haya una selección adecuada de instrumentos en cada nivel.

## Destacados

**(1)** La metodología de Kirkpatrick es eficaz para evaluar los distintos niveles de formación de los profesionales de enfermería.

**(2)** La metodología apoya la elección de instrumentos de medida para cada nivel.

**(3)** Los cuatro niveles o una combinación de los tres primeros fueron los más utilizados para evaluar la formación.

**(4)** Los cuatro niveles o una combinación de los tres primeros fueron los más frequentes para evaluar la formación.

**(5)** La evaluación de los resultados en las prácticas organizativas es el nivel más desafiante.

## Introducción

La formación del personal sanitario se ha incorporado como estrategia de retención del talento en la gestión de los recursos humanos. Las prácticas de formación y desarrollo en el lugar de trabajo se describen como procesos dinámicos y continuos cuyo objetivo es promover avances sociales, aumentar la resolución y fomentar mejores resultados de salud^(1)^.

La formación puede ser una oportunidad para el desarrollo profesional y para aumentar la productividad, la creatividad y la innovación. Estos son los principales aspectos de los servicios de alta calidad y de la eficacia de los equipos^(2)^. La falta de formación puede influir negativamente en la percepción del estrés, aumentar la rotación y el estrés y disminuir el rendimiento laboral^(3)^.

Las estrategias de formación son fundamentales para mejorar los sistemas sanitarios en los países de bajos ingresos^(4)^. El desarrollo de los trabajadores a través de la formación es un indicador crítico de una gestión eficaz^(5)^. En Brasil, la formación también tiene un carácter político, ya que el conocimiento es una parte esencial de la lucha por el poder y un aprendizaje significativo puede conducir a cambios profundos en el sector salud^(6)^.

La evaluación de los programas de formación puede ayudar a los administradores a comprender la eficacia y la sostenibilidad del proceso de aprendizaje para producir cambios a corto o largo plazo. Sin embargo, seleccionar un marco adecuado para la evaluación no es una tarea sencilla y muchas intervenciones se enfrentan a dificultades con niveles bajos o muy bajos de evidencias de los resultados deseados^(4)^.

En 1959, Donald Kirkpatrick ideó y publicó una metodología para ayudar a los directivos a evaluar los resultados de las prácticas de formación y desarrollo de los trabajadores y los sistemas organizativos^(7-8)^, basada en evaluaciones sumativas. El modelo consta de cuatro niveles de evaluación: Reacción, Aprendizaje, Comportamiento y Resultados^(7)^. La reacción (nivel I) abarca las percepciones de los participantes sobre su experiencia de aprendizaje, la estructura del programa, el contenido, los métodos de enseñanza y los aspectos instructivos, como los materiales y la calidad de la enseñanza. El aprendizaje (nivel II) está relacionado con los cambios de actitudes, percepciones, conocimientos y aptitudes. El comportamiento (nivel III) evalúa la transferencia de conocimientos al lugar de trabajo a través de cambios de comportamiento. Por último, el nivel de resultados (nivel IV) evalúa la aplicabilidad de los resultados de la intervención para cambiar la práctica organizativa y mejorar los resultados^(7-8)^.

Los administradores e investigadores deberían considerar detenidamente la adopción de un sistema de evaluación basado en este modelo, ya que no se recomienda la aplicación de niveles aislados y los niveles superiores de evaluación no conducen a resultados más valiosos si se aíslan^(7)^.

El modelo Kirkpatrick ha sido ampliamente aceptado en la comunidad científica para apoyar el desarrollo y la formación en diversos ámbitos, incluido el sanitario^(9-14)^. Varias mejoras han sido descritas por el Grupo Kirkpatrick desde su primera publicación, llegando a un nuevo modelo llamado modelo *New World Kirkpatrick* en 2016^(15)^. A pesar de las mejoras, existen críticas sobre la aplicabilidad del modelo Kirkpatrick^(16-18)^, siendo la principal preocupación la dificultad de implementar los cuatro niveles^(16)^. Estas críticas motivaron esta revisión para asegurar la aplicabilidad del método en enfermería. El objetivo de este estudio es evaluar la evidencia sobre el uso del modelo de Donald Kirkpatrick en la evaluación de la formación de enfermería.

## Métodos

### Tipo de estudio

Utilizamos una revisión bibliográfica integradora, basada en el modelo de Whittemore y Knafl, llevada a cabo en cinco etapas: identificación del problema; búsqueda/criba bibliográfica; evaluación de los datos; análisis de los datos; y presentación de la revisión^(19)^. Elegimos esta estructura metodológica para garantizar un enfoque sistemático de la síntesis de las evidencias. El resultado pretendido es obtener una comprensión global del uso del modelo de Kirkpatrick en enfermería y la identificación de lagunas para futuros estudios^(19)^.

Esta revisión integradora se registró en la plataforma *Open Science Framework* (OSF) el 20 de mayo de 2022, y se puede acceder al protocolo en osf.io/uprv7, con el siguiente DOI 10.17605/OSF.IO/JQ8U9^(20)^.

### Localización

Este análisis integrador se llevó a cabo en el municipio de Thunder Bay, situado en la provincia de Ontario (ON), Canadá.

### Periodo de tiempo

El periodo de estudio fue de septiembre de 2022 a noviembre de 2023.

### Población

Se utilizó la pregunta de revisión: “¿Cuál es la evidencia para utilizar el modelo de Donald Kirkpatrick para evaluar la formación en el lugar de trabajo en enfermería?”. La formulación de la pregunta de investigación se desarrolló utilizando la estrategia PCC, a saber: Población (P): enfermería; Concepto (C): modelo de cuatro niveles de Donald Kirkpatrick; Contexto (C): evaluación de la formación en el lugar de trabajo.

### Criterios de selección

Se incluyeron los estudios que abordaban la pregunta de investigación y se publicaron en inglés, español o portugués entre 2006 y 2023, con contenido completo disponible. El período de tiempo se basó en el año de publicación de la tercera edición del libro “Evaluación de programas de formación”, en el que se actualizaron algunas interpretaciones del modelo tradicional^(7)^. Se excluyeron los estudios que no cumplían los criterios de inclusión o que no describían claramente los instrumentos utilizados. También se eliminaron los duplicados, las revisiones bibliográficas y la literatura gris.

### Definición de la muestra

La búsqueda fue realizada en noviembre de 2022 en Thunder Bay, ON, Canadá, por FMM, y actualizada en agosto de 2023. Se utilizaron las bases de datos de investigación bibliográfica Literatura Latinoamericana y del Caribe en Ciencias de la Salud (LILACS), MEDLINE (vía PubMed) y *Web of Science* (WoS).

Las estrategias de búsqueda se basaron en los Descriptores en Ciencias de la Salud (DeCS) y *Medical Subject Headings* (MeSH). Después de analizar las búsquedas exploratorias, decidimos incluir la palabra clave “Kirkpatrick” para reducir el alcance de la investigación. La estrategia final utilizada fue: (“Education, continuing” OR “Continuous Learning” OR “Learning, Continuous” OR “Lifelong Learning” OR “Learning, Lifelong” OR “Life-Long Learning” OR “Learning, Life-Long” OR “Learnings, Life-Long” OR ’Life Long Learning” OR “Life-Long Learnings” OR “Continuing Education”) AND ((“nurses” OR ’Nurse” OR “Personnel, Nursing” OR “Nursing Personnel” OR “Registered Nurses” OR ’Nurse, Registered” OR “Nurses, Registered” OR “Registered Nurse”) OR (“nursing, team”)) AND (“Kirkpatrick”) para MEDLINE y WoS. Para LILACS, se decidió utilizar únicamente la palabra clave “Kirkpatrick”, teniendo en cuenta la especificidad del tema y el número de estudios recuperados en la estrategia original.

Tras la búsqueda en las bases de datos, los archivos generados en formato BIBTXT o RIS se exportaron al gestor de revisiones Rayyan®^(21)^, que apoyó el cribado inicial de resúmenes y títulos mediante un proceso semiautomático. La selección de los artículos fue realizada en dos fases ciegas por dos investigadores diferentes (FMM y BVS); el primer cribado se completó con la lectura de los títulos y resúmenes de los documentos recuperados; el segundo cribado implicó una revisión completa. Se discutieron los conflictos hasta llegar a un consenso. Un tercer investigador (VAM) estaba disponible para decidir sobre cualquier conflicto persistente.

### Recopilación de datos

Los datos se extrajeron mediante una matriz analítica construida en Microsoft Excel® que contenía información sobre las características de los estudios (autores, año de publicación, país de publicación, objetivo, tipo de estudio, muestra y resultados) e información sobre el uso del modelo de Kirkpatrick (niveles utilizados, elementos, tiempo, criterios de evaluación y medidas).

### Procesamiento y análisis de los datos

Los datos se analizaron mediante reducción y comparación para identificar patrones, temas o relaciones^(19)^. Posteriormente, se realizó un análisis descriptivo basado en el marco teórico-conceptual adoptado, considerando los preceptos del modelo de Donald Kirkpatrick^(7)^.

Esta revisión siguió las directrices del *Preferred Reporting Items for Systematic Reviews and Meta-Analyses* (PRISMA) para la elaboración de informes de revisiones sistemáticas^(22)^. Las directrices PRISMA fueron diseñadas para revisiones sistemáticas de estudios que evalúan los efectos de intervenciones sanitarias; sin embargo, los puntos de la lista de verificación son aplicables a informes de revisiones sistemáticas en otras áreas o que evalúan otros tipos de intervenciones^(22)^. Así, todas las secciones de las directrices PRISMA se utilizaron para guiar el informe de la revisión, excepto los temas no aplicables al enfoque de esta revisión, incluidos los análisis del riesgo de sesgo, la determinación de las medidas del efecto y los análisis de las posibles causas de heterogeneidad. La efectividad del método Kirkpatrick se analizó a través de los resultados de efectividad del entrenamiento mostrados en cada artículo incluido en la muestra seleccionada para este estudio. Para ello, se tuvo en cuenta el diseño de la formación y la utilización de los niveles de Kirkpatrick, la configuración del instrumento utilizado, el momento de la evaluación y otros aspectos.

## Resultados

Se recuperaron 108 artículos y se incluyeron 13 en la muestra final (Figura 1).

La Figura 2 muestra los 13 artículos caracterizados por país, año de publicación, objetivo y métodos.

**Figura 1 -:** Diagrama de flujo PRISMA^(22)^ para selección de los estudios. Thunder Bay, ON, Canadá, 2023

**Figura 2 -:** Caracterización de la muestra (n = 13). Thunder Bay, ON, Canadá, 2023

*WB = *Web-based*(virtual); ^†^RN = *Registered nurse*(enfermero registrado); ^‡^EN = *Enrolled nurse*(enfermeiro reclutado); ^§^CIPE = *Continuing interprofessional education*(Educación interprofesional continuada); ^||^VJC = *Virtual journal club’s*; ^¶^PCC = *Person-centered and competency-based program*(Programa centrado en la persona y basado en las competencias); **FF = *Face-to-face*(cara a cara); ^††^CEC = *Continuing education course*(Curso de educación continuada); ^‡‡^GI = Grupo de intervención; ^§§^GC = Grupo control.

El modelo Kirkpatrick ha sido útil en cursos de formación, formación continua y actualización, así como en programas de especialización con intervenciones centradas en el individuo y basadas en el trabajo en equipo. La Figura 3 muestra el uso del modelo Kirkpatrick de cuatro niveles para evaluar los resultados de la formación:

El modelo de Kirkpatrick fue eficaz en la evaluación de todas las formaciones identificadas, considerando los resultados mensurables y prolíficos de las muestras por nivel en los diferentes tipos de formación. Se observó consistencia en el cumplimiento de las directrices, demostrando su versatilidad y flexibilidad.

**Figura 3 -:** Utilización del modelo de cuatro niveles de Kirkpatrick para evaluar los resultados de la formación (n = 13). Thunder Bay, ON, Canadá, 2023

Brasil fue el único país con más de una publicación^(25-26,28-29)^. Fueron encontrados abordajes cuasiexperimentales^(10,27-28,30-32)^ seguidos de investigación cualitativa^(24,26,33)^, investigación con métodos mixtos^(23,25,29)^ e un estudio experimental^(34)^.

La mayoría de los estudios utilizaron los cuatro niveles del modelo de Kirkpatrick^(10,25,28,30,32-33)^ o una combinación de los tres primeros niveles^(23,26,29,31,34)^. Un artículo evaluó la intervención con una combinación de los niveles I y II^(27)^ y otro con una combinación de los niveles III y IV^(24)^.

La Figura 4 muestra una cronología de la aplicación de estos niveles.

**Figura 4 -:** Se recogieron varios datos antes y después de la intervención utilizando los cuatro niveles del modelo de Kirkpatrick (n = 13). Thunder Bay, ON, Canadá, 2023

Dos estudios^(28,33)^ no identificaron el calendario de recogida de datos. Solo describieron la recogida de datos transversal, considerando la disponibilidad de la intervención durante un periodo de tiempo, como “desde 2014”^(28)^ o “entre 2008 y 2011”^(33)^. Ninguno de los estudios contaba con proyectos de seguimiento explícitos, aunque algunos de ellos recogieron datos mucho tiempo después de la intervención^(10,24-25,32)^. En uno de ellos, los autores citaron la recogida previa de datos tras la intervención, pero no presentaron ni compararon los datos, ni caracterizaron un estudio de seguimiento^(24)^.

Se han utilizado diferentes instrumentos para evaluar las intervenciones, en función de la especificidad de la investigación; sin embargo, no se encontró ningún enfoque principal o instrumento estándar para evaluar cualquier nivel del método de Kirkpatrick. Teniendo en cuenta la relevancia de los instrumentos de medida para los cuatro niveles del modelo de Kirkpatrick y la falta de consenso sobre la mejor forma de hacerlo (y esta es la riqueza del modelo - se puede personalizar según el estudio), se decidió sintetizar los instrumentos que se utilizaron en cada estudio seleccionado para ayudar a futuros enfermeros en este tema.

Los instrumentos cuantitativos fueron los más comunes. La Tabla 1 muestra los instrumentos cuantitativos utilizados para evaluar los programas de formación, sus medidas (ítems y rango) y si el estudio describía alguna fuente de fiabilidad mediante el α de Cronbach.

**Tabla 1 -:** Instrumentos cuantitativos aplicados para evaluar el modelo de cuatro niveles de Kirkpatrick (n = 13). Thunder Bay, ON, Canadá, 2023

**Nivel: Descripción del instrumento**	Ítem	**Rango**	α*
I: escala Likert de cinco puntos para la satisfacción de los estudiantes^(23)^	5	1-5	n/s^†^
I: Cuestionario con escala Likert de cinco puntos: “material y programa de estudio”, “tareas”, “tutoría” y “estructura de apoyo al aprendizaje”^(25)^	4	1-5	n/s^†^
I: Herramienta de evaluación de la reacción que incluye aspectos exógenos (por ejemplo, acceso a Internet) y endógenos (por ejemplo, material) para cada módulo y para todo el curso^(29)^	9	1-4	n/s^†^
I: NOIC^‡^ con una escala Likert de 5 puntos^(10)^	21	1-5	0,94
I: ABC-SAT^§^, dominios: a = afectivo, b = conductual, c = cognitivo^(27)^	a = 3	0-12	0,60
	b = 2	0-8	0,66
	c = 6	0-24	0,83
I: Declaraciones en escala Likert de cinco puntos relativas a la satisfacción, el potencial ideal de aprendizaje y la evitación de malos sentimientos^(28)^	3	1-5	n/s^†^
I: Cuestionario con escala Likert de cinco puntos, dominios: c = contenido, t = profesores, f = instalaciones^(30)^	c = 5	1-5	0,87
	t = 4		
	f = 3		
I: Encuesta con escala Likert de cinco puntos relativa a la usabilidad, el formato, las oportunidades de debate, la participación y la adecuación del tiempo^(31)^	6	1-5	n/s^†^
I: IMMS^||(32)^	4	1-5	0,79
I: Cuestionario de satisfacción autodeclarada, dominios c = “contenido y su relevancia” y s = “apoyo y facilitación”^(34)^	c = 35	0-35	0,92
	s = 15	0-15	0,77
II: Prueba de confianza y conocimientos educativos de 10 preguntas^(23)^	10	5-50	0,92
II: Cuatro pruebas de procedimiento (una por cada módulo de aprendizaje)^(25)^	n/s^†^	0-10	n/s^†^
II: Cuestionario de autoeficacia para la innovación^(10)^	8	1-5	0,86
II: Prueba escrita autoadministrada de conocimientos sobre temas clave de la intervención, cada una con cuatro respuestas posibles^(27)^	9	0-18	n/s^†^
II: Declaraciones en escala Likert de cinco puntos: “(...) me sirvió para aumentar mis conocimientos teóricos” y “(...) me sirvió para aumentar mi capacidad para resolver problemas clínicos”^(28)^	2	1-5	n/s^†^
II: Cuestionario de sensibilización^(30)^	15	0-15	0,87
II: Test cerrado con cinco preguntas (dos tenían más de una respuesta correcta)^(31)^	5	0-5	n/s^†^
II: Cuestionario de metacognición^(32)^	30	n/s^†^	n/s^†^
II: Prueba de conocimientos de 1 hora de duración^(34)^	30	0-30	n/s^†^
II: Presentación de una tarea que requería la descripción de un programa de ejercicios adaptado a un escenario hipotético de un cliente^(34)^	1	0-100	n/s^†^
III: Cuestionario de autoinforme sobre el comportamiento en relación con los protocolos de seguridad del paciente^(25)^	9	1-5	n/s^†^
III: NIBS¶^(10)^	10	1-5	0,90
III: Dos afirmaciones cerradas sobre la reducción de la ansiedad/estrés y la capacidad para resolver un problema/situación clínica^(28)^	2	1-5	n/s^†^
III: Cuestionario de rendimiento^(30)^	15	0-15	0,78
III: Cuestionario de transferencia de conocimientos en el lugar de trabajo^(32)^	14	1-5	0,94
III: Cuestionario de satisfacción autodeclarada, dominio b = “cambio en el comportamiento clínico”^(34)^	b = 30	0-30	0,84
IV: Cuestionario sobre el acuerdo con la aplicación de cinco objetivos para la seguridad del paciente en el lugar de trabajo después del curso^(25)^	6	1-5	n/s^†^
IV: Escala de Capacidad de Innovación del Equipo Clínico de Enfermería^(10)^	41	0-205	0,82
IV: Cuestionario de exposición a objetos punzantes, sangre y fluidos corporales^(30)^	53	n/e^†^	0,89

*α = α de Cronbach; ^†^n/e = No especificado; ^‡^NOIC = *The Nurse Organizational Innovation Climate Scale* (Escala de Clima de Innovación Organizativa del Equipo de Enfermería); ^§^ABC-SAT = *The ‘Affective - Behavioral - Cognitive - Satisfaction Questionnaire’* (Cuestionario de Satisfacción ‘Afectivo - Conductual – Cognitivo’); ^||^IMMS = *The Instructional Materials Motivation Survey* (Encuesta de Motivación del Material Didáctico); ^¶^NIBS = *Nurse Innovation Behaviour Scale* (Escala de Comportamiento Innovador del Personal de Enfermería)

Se encontró una gran variabilidad de instrumentos en lo que respecta a los enfoques cuantitativos. El nivel I se evaluó principalmente mediante una escala Likert de cinco puntos para ítems como satisfacción^(23,25,27-28,30,32,34)^, inmersión^(28,31)^, clima^(10)^, relevancia y confianza^(32)^. En el nivel II se utilizaron enfoques de conocimiento y sensibilización. Los más comunes fueron las pruebas de aprendizaje cerradas^(25,27,29-31,34)^, seguidas de cuestionarios de autoevaluación^(10,23,32)^. Las medidas cuantitativas de los niveles III y IV incluían las percepciones de los estudiantes sobre la transferencia de conocimientos al lugar de trabajo mediante cuestionarios cerrados^(10,25,28,30,32-34)^ o escalas^(10)^.

Aunque menos frecuente que el cuantitativo, el uso de un enfoque cualitativo se encontró en el nivel conductual^(23-24,26,29,31,33)^ seguido de la reacción^(26,29,33)^, resultados^(24,28,33)^ y el aprendizaje^(26,33)^. Siete estudios^(23-24,26,28-29,31,33)^ utilizaron instrumentos cualitativos para evaluar los programas de formación, pero sólo dos de ellos^(24,26)^ lo hicieron sin una parte cuantitativa. Se identificaron entrevistas semiestructuradas^(23-24,26)^, cuestionarios con preguntas abiertas^(28,33)^, análisis descriptivo de palabras clave en proyectos de estudiantes^(29)^, investigación cualitativa^(31)^, análisis cualitativo basado en las narraciones de los participantes^(29)^ y adherencia tras el primer mes de la intervención^(29)^. El tipo de análisis cualitativo más prevalente fue el análisis temático^(24,26,28-29)^. La mayoría de los estudios no describieron la fiabilidad o validez de los instrumentos cualitativos, con la excepción de Koto-Shimada^(24)^, que realizó una prueba piloto. También se han descrito otras medidas relacionadas con el trabajo, como las historias clínicas y la información sobre los pacientes^(32)^ o el cuestionario de exposición autodeclarada a lesiones por pinchazo de aguja^(30)^.

La elección de una herramienta de medición es fundamental para evaluar la eficacia de cualquier intervención, especialmente cuando se trata de formación. Este paso está incorporado en el modelo de Kirkpatrick y debe ser cuidadosamente seleccionado por los enfermeros para cualificar el análisis de cada nivel. Cuanto más precisa sea la herramienta (es decir, más acorde con el objeto de estudio), mayores serán las posibilidades de obtener una evaluación fiable. Cuanto más precisa sea la herramienta (es decir, más acorde con el objeto de estudio), mayores serán las posibilidades de una evaluación fiable.

## Discusión

Esta revisión mostró que el modelo de Kirkpatrick ha sido ampliamente utilizado para evaluar la formación de enfermeros y también en otras áreas de la atención sanitaria^(11)^. Nuestra muestra retratada en este estudio indicó coherencia en el cumplimiento de las directrices y los principales resultados de la formación indican que este modelo es útil para evaluar la eficacia, con resultados positivos en cada nivel, en diferentes tipos de intervenciones de formación.

El proceso de evaluación convergió con la orientación del modelo, que se centra en los resultados de los participantes^(7)^. Sin embargo, un estudio se dirigió a los directivos de los participantes y surgió una visión complementaria, como se ejemplifica en la cita: “los enfermeros no sintieron el cambio de ambiente y actitudes de la misma manera que habían observado los directivos de enfermería”^(33)^. La inclusión de la perspectiva del liderazgo puede ser positiva, sobre todo para obtener información sobre la transferencia de conocimientos al lugar de trabajo (nivel III) o los cambios en la práctica o los resultados organizativos (nivel IV), teniendo en cuenta su función supervisora.

El tipo de información de referencia más común fue en el nivel de Aprendizaje^(10,23,27,30,32)^. Los niveles de Comportamiento y Resultados fueron los más divergentes en relación con el momento. No hay directrices en el modelo de Kirkpatrick sobre el mejor momento para evaluar cada nivel^(7,15)^ y no podemos apoyar un momento más común o asertivo para la recogida de datos, ya que ninguno de los estudios converge en el mejor momento. Sin embargo, es importante tener en cuenta que los programas de formación esperan resultados a corto, medio y largo plazo. Por lo tanto, seleccionar un momento específico (por ejemplo, antes, durante, poco después, más tiempo después) o un periodo (por ejemplo, seguimientos) es esencial para una evaluación exhaustiva que tenga en cuenta que la sensación de reacción disminuirá con el tiempo y los resultados más importantes aparecerán con el tiempo^(8)^.

El modelo de Kirkpatrick es una estructura flexible y ajustable que incorpora diferentes herramientas para evaluar los resultados de la formación (Reacción, Aprendizaje, Comportamiento y Resultados), lo que refuerza su poder, ya que las realidades, la formación y los contextos de cada organización son completamente diferentes. Sin embargo, precisamente por no indicar un estándar metodológico específico, los autores deben ser cuidadosos para garantizar la validez y fiabilidad de la información recogida^(17)^.

Se observó que tanto el número de estudios como la diversidad de instrumentos disminuían a medida que aumentaba el nivel de evaluación. La revisión bibliográfica indica que el nivel “Resultados” es el más difícil de alcanzar. Se reconoce que el nivel IV “podría identificar el valor añadido para la sociedad de un determinado programa educativo, ya que permite evaluar no sólo la aplicación de los proyectos de intervención en la práctica de los profesionales de salud, sino también los resultados de su utilización en un contexto local”^(29)^. Sin embargo, la mitad de las muestras de esta revisión no informaron de la medición de este nivel. La razón más común comunicada para excluir el nivel IV fue la necesidad de períodos de seguimiento a medio y largo plazo^(26)^ y la complejidad de relacionar los resultados con el programa de formación, distinguiéndolos de otros muchos factores que podrían afectar a los resultados^(31)^. La evaluación de resultados (nivel IV) es la más desafiante porque debe considerar la transformación o el impacto de la formación en la práctica organizativa, lo que requiere otro tipo de instrumentos, información organizativa y el punto de vista de otras partes interesadas, así como más tiempo. En este sentido, se encontraron datos similares sobre la relevancia percibida de los niveles III y IV y la dificultad de implementar estos niveles^(17)^.

Existen algunas críticas a la rigidez de este modelo, que puede llevar a no considerar aspectos esenciales de la evaluación, como la visión formativa^(16,18)^. Sólo un estudio informó de la recogida de datos en cada módulo y al final del curso a nivel de reacciones^(29)^. Ninguna otra experiencia fue identificada en el seguimiento. Esta evidencia muestra que, a pesar de la diversidad en la perspectiva de las herramientas de evaluación de resultados, especialmente con el uso de enfoques cualitativos para capturar la perspectiva de los estudiantes, todas las experiencias reportadas fueron en una visión sumativa o transversal.

Los instructores no están utilizando sólo los niveles más altos, que es una preocupación de las críticas al modelo de Kirkpatrick^(16-18)^, como se ha señalado.

Seis artículos consiguieron alcanzar el nivel cuatro y otros cinco utilizaron una combinación de los tres primeros niveles, lo que representa el 85% de la muestra. Esto indica que los instructores de enfermería intentan utilizar los cuatro niveles del modelo para recopilar información sobre los resultados, a pesar del reto que supone el nivel de estos. Esto reafirma el compromiso de los investigadores con la recomendación de utilizar un modelo completo para obtener una mayor precisión^(7-8)^.

La relevancia de esta revisión radica en que resume el reconocimiento de la operacionalización del modelo.

Un amplio análisis bibliométrico descubrió que el modelo de Kirkpatrick era un tema de creciente interés a partir de la década de 2000, incluyendo el 20,7% de su muestra como algún tipo de publicación de revisión bibliográfica. Sin embargo, la mayoría de los estudios tenían como objetivo criticar el modelo o identificar sus beneficios, y se encontró poca evidencia de revisión sobre los tipos y la caracterización de las herramientas de evaluación y su aplicación en un área específica, como la enfermería^(11)^.

Este estudio tiene limitaciones metodológicas. Los diversos proyectos de investigación de las publicaciones incluidas imposibilitaron la obtención de pruebas mediante técnicas de metaanálisis o metasíntesis. Teniendo en cuenta la naturaleza de la revisión integradora, el objetivo no era evaluar críticamente la calidad de los estudios, y por esta razón la calidad de los estudios no se utilizó como criterio de inclusión.

Alentamos futuras investigaciones para identificar los mejores constructos para evaluar cada nivel, ampliar la aplicación del nivel IV y promover la discusión científica sobre la orientación metodológica para componer la cadena de evidencias entre los niveles, como propone el modelo *New World Kirkpatrick* ^(15)^. Además, nuestra revisión de la literatura identificó limitados estudios longitudinales con seguimientos consecutivos para la recolección de datos, lo que es altamente recomendable para la obtención de evidencias de alto nivel en relación con las vías causales.

## Conclusión

El modelo de cuatro niveles de Kirkpatrick es una elección adecuada para adaptar los instrumentos de evaluación al proyecto de formación. La selección de un conjunto fiable de instrumentos es clave para cualificar el análisis de cada nivel, siendo las escalas de Likert la opción más común para los formadores. Este modelo es prolífico para evaluar la formación en enfermería, pero requiere una elección cuidadosa de los instrumentos para cada nivel. Se identificaron muchas herramientas de medición cualitativas y cuantitativas que podrían ser útiles para profesionales y académicos en futuras evaluaciones e investigaciones.

# Translation: O emprego da metodologia de Kirkpatrick para avaliar a formação em enfermagem: uma revisão integrativa*

**DOI:** 10.1590/1518-8345.7250.4432

**Authors:** Fernanda Maria de Miranda, Bruna Vasconcelos dos Santos, Vicki Leigh Kristman, Vivian Aline Mininel

**Affiliations:** 1 Universidade Federal de São Carlos, São Carlos, SP, Brasil; 2 Bolsista da Coordenação de Aperfeiçoamento de Pessoal de Nível Superior (CAPES), Brasil; 3 Lakehead University, Department of Health Sciences, Thunder Bay, ON, Canada; 4 Universidade Federal de São Carlos, Departamento de Enfermagem, São Carlos, SP, Brasil

**Keywords:** Educação Continuada, Enfermeiras e Enfermeiros, Avaliação de Eficácia-Efetividade de Intervenções, Revisão, Método, Capacitação em Serviço

## Abstract

avaliar as evidências sobre o uso do modelo de Donald Kirkpatrick na avaliação da formação em enfermagem.

revisão integrativa da literatura nas bases de dados Literatura Latino-Americana e do Caribe em Ciências da Saúde, *Medical Literature Analysis and Retrieval System* e *Web of Science*. Foram incluídos estudos que respondem à questão de revisão “Quais são as evidências da utilização do modelo de Donald Kirkpatrick para avaliar o treinamento no ambiente de trabalho da enfermagem?” publicados em português, inglês ou espanhol.

dos 108 estudos recuperados, treze foram incluídos. A maioria avaliou os quatro níveis propostos no modelo (reação, aprendizagem, comportamento e resultados) ou, pelo menos, uma combinação dos três primeiros. Foram utilizados diferentes instrumentos para avaliar a formação em enfermagem, principalmente em abordagens quantitativas para os níveis de reação e aprendizagem e qualitativas para os níveis de comportamento e resultados. Esta abordagem evidencia a flexibilidade do modelo e a importância da escolha de um conjunto confiável de instrumentos, fundamental para qualificar a análise em cada nível.

o modelo de Kirkpatrick tem sido utilizado em todo o mundo para avaliar a formação na área da enfermagem e tem se revelado adequado para o objetivo, desde que haja uma seleção adequada de instrumentos em cada nível.

## Destaques

**(1)** A metodologia de Kirkpatrick é eficaz na avaliação de vários níveis de formação em enfermagem.

**(2)** A metodologia suporta a escolha de instrumentos de medida para cada nível.

**(3)** Os quatro níveis ou uma combinação dos três primeiros foram os mais utilizados para avaliar a formação.

**(4)** Os quatro níveis ou uma combinação dos três primeiros foram os mais comuns para avaliar a formação.

**(5)** A avaliação dos resultados nas práticas organizacionais é o nível mais desafiante.

## Introdução

A formação de pessoal de saúde tem sido incorporada como uma estratégia de retenção de talentos na gestão de recursos humanos. As práticas de formação e desenvolvimento no local de trabalho são descritas como processos dinâmicos e contínuos que têm por objetivo promover avanços sociais, aumentar a resolutividade e fomentar melhores resultados na saúde^(1)^.

O treinamento pode ser uma oportunidade de desenvolvimento profissional e de aumento da produtividade, criatividade e inovação. Estes são os principais aspectos dos serviços de alta qualidade e da eficácia das equipes^(2)^. A falta de treinamento pode influenciar negativamente a percepção do estresse, aumentar a rotatividade e o estresse e deteriorar o desempenho no trabalho^(3)^.

As estratégias de treinamento são fundamentais para melhorar os sistemas de saúde em países de baixa renda^(4)^. O desenvolvimento dos trabalhadores por meio de treinamento é um indicador crítico de uma gestão eficaz^(5)^. No Brasil, o treinamento também tem caráter político, uma vez que o conhecimento é parte essencial da luta pelo poder e a aprendizagem significativa pode levar a mudanças profundas no setor de saúde^(6)^.

A avaliação dos programas de treinamento pode ajudar os administradores a entender a eficácia e a sustentabilidade do processo de aprendizagem para produzir mudanças a curto ou longo prazo. No entanto, a seleção de uma estrutura adequada para a avaliação não é uma tarefa simples e muitas intervenções enfrentam dificuldades com níveis baixos ou muito baixos de evidência para os resultados almejados^(4)^.

Em 1959, Donald Kirkpatrick idealizou e publicou uma metodologia para ajudar os administradores a avaliar os resultados das práticas de treinamento e desenvolvimento entre os trabalhadores e os sistemas organizacionais^(7-8)^, com base em avaliações somativas. O modelo é composto por quatro níveis de avaliação: Reação, Aprendizagem, Comportamento e Resultados^(7)^. A reação (nível I) abrange as percepções do participante sobre sua experiência de aprendizagem, a estrutura do programa, o conteúdo, os métodos de ensino e os aspectos instrucionais, como os materiais e a qualidade da instrução. A aprendizagem (nível II) está relacionada a mudanças de atitudes, percepções, conhecimentos e habilidades. O comportamento (nível III) avalia a transferência de conhecimento para o local de trabalho por meio de mudanças comportamentais. Finalmente, o nível de resultados (nível IV) avalia a aplicabilidade dos resultados da intervenção para mudar a prática organizacional e melhorar os resultados^(7-8)^.

Os administradores e pesquisadores devem considerar cuidadosamente a adoção de um sistema de avaliação baseado neste modelo, pois a implementação de níveis isolados não é recomendada e níveis mais altos de avaliação não levam a resultados mais valiosos se forem isolados^(7)^.

O modelo Kirkpatrick tem sido amplamente aceito na comunidade científica para apoiar o desenvolvimento e o treinamento em diversas áreas, inclusive na saúde^(9-14)^. Diversas melhorias foram descritas pelo Grupo Kirkpatrick desde a primeira publicação, chegando a um novo modelo denominado modelo *New World Kirkpatrick*em 2016^(15)^. Apesar dos aprimoramentos, há críticas quanto à aplicabilidade do modelo de Kirkpatrick^(16-18)^, sendo a principal preocupação a dificuldade de implementar todos os quatro níveis^(16)^. Estas críticas motivaram esta revisão para garantir a aplicabilidade do método na enfermagem. O objetivo deste estudo é avaliar as evidências sobre o uso do modelo de Donald Kirkpatrick na avaliação do treinamento de enfermagem.

## Método

### Tipo de estudo

Uma revisão integrativa da literatura foi utilizada, com base no modelo de Whittemore e Knafl, realizada em cinco estágios: identificação do problema; busca/triagem da literatura; avaliação dos dados; análise dos dados; e apresentação da revisão^(19)^. Esta estrutura metodológica foi escolhida para garantir uma abordagem sistemática da síntese de evidências. O resultado pretendido foi obter uma compreensão abrangente do uso do modelo de Kirkpatrick na enfermagem e a identificação de lacunas para estudos futuros^(19)^.

Esta revisão integrativa foi registrada na plataforma *Open Science Framework*(OSF) em 20 de maio de 2022, e o protocolo pode ser acessado em osf.io/uprv7, com o seguinte DOI 10.17605/OSF.IO/JQ8U9^(20)^.

### Local

Esta análise integrativa foi realizada no município de Thunder Bay, localizado na província de Ontario (ON), Canadá.

### Período

O período de estudo foi de setembro 2022 a novembro 2023.

### População

A pergunta da revisão empregada foi: “Quais são as evidências do uso do modelo de Donald Kirkpatrick para avaliar o treinamento no local de trabalho da enfermagem?” A formulação da pergunta de pesquisa foi desenvolvida a partir da estratégia PCC, a saber: População (P): enfermagem; Conceito (C): Modelo de quatro níveis de Donald Kirkpatrick; Contexto (C): avaliação do treinamento no local de trabalho.

### Critérios de seleção

Os estudos foram incluídos se abordassem a questão da pesquisa e fossem publicados em inglês, espanhol ou português entre 2006 e 2023, com conteúdo completo disponível. O período de tempo baseou-se no ano de publicação da terceira edição do livro “*Evaluating training programs*”, em que algumas interpretações do modelo tradicional foram atualizadas^(7)^. Foram excluídos os estudos que não atendiam aos critérios de inclusão ou que não descreviam claramente os instrumentos utilizados. Também foram removidas as duplicatas, revisões de literatura e literatura cinzenta.

### Definição da amostra

A busca foi realizada em novembro de 2022 em Thunder Bay, ON, Canadá, pela FMM, e atualizada em agosto de 2023. Foram usadas as bases de dados de pesquisa bibliográfica Literatura Latino-Americana e do Caribe em Ciências da Saúde (LILACS), MEDLINE (via PubMed) e *Web of Science*(WoS).

As estratégias de busca foram baseadas nos Descritores em Ciências da Saúde (DeCS) e nos *Medical Subject Headings*(MeSH). Após a análise das pesquisas exploratórias, decidiu-se incluir a palavra-chave “Kirkpatrick” para reduzir o escopo da pesquisa. A estratégia final usada foi: (“Education, continuing” OR “Continuous Learning” OR “Learning, Continuous” OR “Lifelong Learning” OR “Learning, Lifelong” OR “Life-Long Learning” OR “Learning, Life-Long” OR “Learnings, Life-Long” OR ’Life Long Learning” OR “Life-Long Learnings” OR “Continuing Education”) AND ((“nurses” OR ’Nurse” OR “Personnel, Nursing” OR “Nursing Personnel” OR “Registered Nurses” OR ’Nurse, Registered” OR “Nurses, Registered” OR “Registered Nurse”) OR (“nursing, team”)) AND (“Kirkpatrick”) para MEDLINE e WoS. Para LILACS, foi decidido usar apenas a palavra-chave “Kirkpatrick”, considerando a especificidade do tema e a quantidade de estudos recuperados na estratégia original.

Após a pesquisa nas bases de dados, os arquivos gerados no formato BIBTXT ou RIS foram exportados para o gerenciador de revisão Rayyan^®^^(21)^, que apoiou a triagem inicial de resumos e títulos usando um processo semiautomático. A seleção de artigos foi realizada em duas etapas cegas por dois pesquisadores diferentes (FMM e BVS); a primeira triagem foi concluída com a leitura dos títulos e resumos dos documentos recuperados; a segunda triagem envolveu uma revisão completa. Os conflitos foram discutidos até que se chegasse a um consenso. Um terceiro pesquisador (VAM) estava disponível para decidir sobre qualquer conflito persistente.

### Coleta de dados

Os dados foram extraídos por meio de uma matriz analítica construída no *Microsoft Excel* ^®^ contendo informações sobre as características dos estudos (autores, ano de publicação, país de publicação, objetivo, tipo de estudo, amostra e resultados) e informações sobre o uso do modelo de Kirkpatrick (níveis utilizados, elementos, tempo, critérios de avaliação e medidas).

### Tratamento e análise de dados

Os dados foram analisados por meio de redução e comparação para identificar padrões, temas ou relações^(19)^. Posteriormente, foi realizada uma análise descritiva com base no referencial teórico-conceitual adotado, considerando os preceitos do modelo de Donald Kirkpatrick^(7)^.

Esta revisão seguiu as diretrizes do *Preferred Reporting Items for Systematic Reviews and Meta-Analyses*(PRISMA) para relatar revisões sistemáticas^(22)^. As diretrizes PRISMA foram projetadas para revisões sistemáticas de estudos que avaliam os efeitos de intervenções de saúde; no entanto, os itens da lista de verificação são aplicáveis a relatórios de revisões sistemáticas em outras áreas ou que avaliam outros tipos de intervenções^(22)^. Assim, todas as seções das diretrizes PRISMA foram usadas para orientar o relatório da revisão, exceto os tópicos não aplicáveis à abordagem desta revisão, incluindo análises do risco de viés, determinação de medidas de efeito e análises de possíveis causas de heterogeneidade. Foi analisada a eficácia do método Kirkpatrick por meio dos resultados de eficácia do treinamento mostrados em cada artigo incluído na amostra selecionada para este estudo. Para isso, foram considerados o projeto do treinamento e o uso dos níveis de Kirkpatrick, a configuração do instrumento utilizado, o momento da avaliação e outros aspectos.

## Resultados

Foram recuperados 108 artigos e 13 foram incluídos na amostra final (Figura 1).

A Figura 2mostra os 13 artigos caracterizados por país, ano de publicação, objetivo e métodos.

**Figura 1 -:** Diagrama de fluxo PRISMA^(22)^ para seleção dos estudos. Thunder Bay, ON, Canadá, 2023

**Figura 2 -:** Caracterização da amostra (n = 13). Thunder Bay, ON, Canadá, 2023

*CIPE = *Continuing interprofessional education*(Educação interprofissional contínua); ^†^UNA-SUS/UERJ = Universidade Aberta do Sistema Único de Saúde/Universidade Estadual do Rio de Janeiro; ^‡^GI = Grupo de intervenção; ^§^GC = Grupo controle; ^||^VJC = *Virtual journal clubs*; ^¶^WB = *Web-based*(virtual); **FF = *Face-to-face*(presencial)

O modelo Kirkpatrick tem sido útil em cursos de treinamento, educação continuada e atualização, bem como em programas de especialização com intervenções centradas no indivíduo e baseadas em equipes. A Figura 3mostra o uso do modelo Kirkpatrick de quatro níveis para avaliar os resultados do treinamento:

**Figura 3 -:** Uso do modelo Kirkpatrick de quatro níveis para avaliar os resultados do treinamento (n = 13). Thunder Bay, ON, Canadá, 2023

*WB = *Web-based*(virtual); ^†^RN = *Registered nurse*(enfermeiro registrada); ^‡^EN = *Enrolled nurse*(enfermeiro recrutado); ^§^CIPE = *Continuing interprofessional education*(Educação interprofissional contínua); ^||^VJC = *Virtual journal club’s*; ^¶^PCC = *Person-centered and competency-based program*(Programa centrado na pessoa e baseado em competências); **FF = *Face-to-face*(presencial); ^††^CEC = *Continuing education course*(Curso de educação continuada); ^‡‡^GI = Grupo de intervenção; ^§§^GC = Grupo controle

O modelo de Kirkpatrick foi eficaz na avaliação de todos os treinamentos identificados, considerando os resultados mensuráveis e prolíficos das amostras por nível em diferentes tipos de treinamento. Foi observada consistência no cumprimento das diretrizes, demonstrando sua versatilidade e flexibilidade.

O Brasil foi o único país com mais de uma publicação^(25-26,28-29)^. Foram encontradas abordagens quase-experimentais^(10,27-28,30-32)^ seguidas de pesquisas qualitativas^(24,26,33)^, pesquisas com métodos mistos^(23,25,29)^ e um estudo experimental^(34)^.

A maioria dos estudos usou todos os quatro níveis do modelo de Kirkpatrick^(10,25,28,30,32-33)^ ou uma combinação dos três primeiros níveis^(23,26,29,31,34)^. Um artigo avaliou a intervenção com uma combinação dos níveis I e II^(27)^ e outro com uma combinação dos níveis III e IV^(24)^.

A Figura 4mostra uma linha do tempo para a implementação destes níveis.

**Figura 4 -:** Vários pontos de dados foram coletados antes e depois da intervenção usando os quatro níveis do modelo de Kirkpatrick (n = 13). Thunder Bay, ON, Canadá, 2023

Dois estudos^(28,33)^ não identificaram a linha do tempo para a coleta de dados. Eles apenas descreveram que houve coleta de dados transversais, considerando a disponibilidade da intervenção por um período de tempo, como “desde 2014”^(28)^ ou “entre 2008 e 2011”^(33)^. Nenhum dos estudos tinha projetos de acompanhamento explícitos, embora alguns deles tenham coletado dados muito tempo após a intervenção^(10,24-25,32)^. Em um deles, os autores citaram uma coleta de dados anterior após a intervenção, mas não apresentaram ou compararam os dados, nem caracterizaram um estudo de acompanhamento^(24)^.

Diferentes instrumentos têm sido utilizados para avaliar intervenções, com base na especificidade da investigação; entretanto, não foi encontrada uma abordagem principal ou um instrumento padrão para avaliar qualquer nível do método de Kirkpatrick. Considerando a relevância dos instrumentos de medição para os quatro níveis do modelo de Kirkpatrick e a falta de consenso sobre a melhor maneira de fazê-lo (e esta é a riqueza do modelo - ele pode ser personalizado de acordo com o estudo), decidiu-se sintetizar os instrumentos que foram usados em cada estudo selecionado para ajudar futuros enfermeiros neste tópico.

Os instrumentos quantitativos foram os mais comuns. A Tabela 1mostra os instrumentos quantitativos usados para avaliar os programas de treinamento, suas medidas (itens e intervalo) e se o estudo descreveu alguma fonte de confiabilidade usando o α de Cronbach.

**Tabela 1 -:** Instrumentos quantitativos aplicados para avaliar o modelo de quatro níveis de Kirkpatrick (n = 13). Thunder Bay, ON, Canadá, 2023

**Nível: Descrição do instrumento**	**Item**	**Intervalo**	α*
I: Escala Likert de cinco pontos para satisfação do estudante^(23)^	5	1-5	n/s^†^
I: Questionário da escala Likert de cinco pontos: “material e programa de estudos”, “tarefas”, “tutoria” e “estrutura de apoio à aprendizagem”^(25)^	4	1-5	n/s^†^
I: Ferramenta de avaliação de reação que inclui aspectos exógenos (por exemplo, acesso à Internet) e aspectos endógenos (por exemplo, material) para cada módulo e para todo o curso^(29)^	9	1-4	n/s^†^
I: NOIC^‡^ com escala Likert de 5 pontos^(10)^	21	1-5	0,94
I: ABC-SAT^§^, domínios: a = afetivo, b = comportamental, c = cognitivo^(27)^	a = 3	0-12	0,60
	b = 2	0-8	0,66
	c = 6	0-24	0,83
I: Declarações em escala Likert de cinco pontos relacionadas à satisfação, potencialidade de aprendizagem ideal e prevenção de sentimentos ruins^(28)^	3	1-5	n/s^†^
I: Questionário em escala Likert de cinco pontos, domínios: c = conteúdo, t = professores, f = instalações^(30)^	c = 5	1-5	0,87
	t = 4		
	f = 3		
I: Pesquisa com escala Likert de cinco pontos relacionada à usabilidade, formato, oportunidades de discussão, participação e adequação do tempo^(31)^	6	1-5	n/s^†^
I: IMMS^||(32)^	4	1-5	0,79
I: Questionário de satisfação autorrelatada, domínios c = “conteúdo e sua relevância” e s = “suporte e facilitação”^(34)^	c = 35	0-35	0,92
	s = 15	0-15	0,77
II: Teste de conhecimento e confiança em educação com 10 perguntas^(23)^	10	5-50	0,92
II: Quatro testes Processuais (um para cada módulo de aprendizagem)^(25)^	n/s^†^	0-10	n/s^†^
II: Questionário de autoeficácia em inovação^(10)^	8	1-5	0,86
II: Teste de conhecimento escrito auto elaborado sobre tópicos-chave da intervenção, cada um com quatro respostas possíveis^(27)^	9	0-18	n/s^†^
II: Declarações da escala Likert de cinco pontos: “(...) foi usado para aumentar meu conhecimento teórico” e ’(...) foi usado para aumentar minha capacidade de resolver problemas clínicos”^(28)^	2	1-5	n/s^†^
II: Questionário de conscientização^(30)^	15	0-15	0,87
II: Teste fechado com cinco perguntas (duas tinham mais de uma resposta correta)^(31)^	5	0-5	n/s^†^
II: Questionário de Metacognição^(32)^	30	n/s^†^	n/s^†^
II: Teste de conhecimento de 1 hora^(34)^	30	0-30	n/s^†^
II: Envio de tarefa que exige a descrição de um programa de exercícios adaptado a um cenário hipotético de cliente^(34)^	1	0-100	n/s^†^
III: Questionário de autorrelato sobre o comportamento em relação aos protocolos de segurança do paciente^(25)^	9	1-5	n/s^†^
III: NIBS^¶(10)^	10	1-5	0,90
III: Duas afirmações fechadas sobre a diminuição da ansiedade/estresse e a capacidade de resolver um problema/situação clínica^(28)^	2	1-5	n/s^†^
III: O questionário de desempenho^(30)^	15	0-15	0,78
III: Questionário de transferência de conhecimento autorrelatado no local de trabalho^(32)^	14	1-5	0,94
III: Questionário de satisfação autorrelatado, domínio b = “mudança no comportamento clínico”^(34)^	b = 30	0-30	0,84
IV: Questionário de concordância sobre a implantação de cinco metas para a segurança do paciente no local de trabalho após o curso^(25)^	6	1-5	n/s^†^
IV: Escala de Capacidade de Inovação da Equipe de Enfermagem Clínica^(10)^	41	0-205	0,82
IV: Questionário de exposição a objetos cortantes, sangue e fluidos corporais^(30)^	53	n/e^†^	0,89

*α = α de Cronbach; ^†^n/e = Não especificado; ^‡^NOIC = *The Nurse Organizational Innovation Climate Scale*(Escala de Clima de Inovação Organizacional da equipe de Enfermagem); ^§^ABC-SAT = *The ‘Affective - Behavioral - Cognitive - Satisfaction Questionnaire’*(Questionário de Satisfação ‘Afetivo - Comportamental – Cognitivo’); ^||^IMMS = *The Instructional Materials Motivation Survey*(Pesquisa de Motivação de Materiais Instrucionais); ^¶^NIBS = *Nurse Innovation Behaviour Scale*(Escala de Comportamento de Inovação do Enfermeiro)

Foi encontrada uma grande variabilidade de instrumentos com relação às abordagens quantitativas. O nível I foi avaliado principalmente por uma escala Likert de cinco pontos para itens como satisfação^(23,25,27-28,30,32,34)^, imersão^(28,31)^, clima^(10)^, relevância e confiança^(32)^. As abordagens de conhecimento e conscientização foram usadas no nível II. O mais comum foram os testes de aprendizagem fechados^(25,27,29-31,34)^, seguidos por questionários autoavaliados^(10,23,32)^. As medidas quantitativas dos níveis III e IV incluíram a percepção dos alunos sobre a transferência de conhecimento para o local de trabalho por meio de questionários fechados^(10,25,28,30,32-34)^ ou escalas^(10)^.

Embora menos frequente do que a quantitativa, o uso de uma abordagem qualitativa foi encontrado no nível comportamental^(23-24,26,29,31,33)^ seguido por reação^(26,29,33)^, resultados^(24,28,33)^ e aprendizagem^(26,33)^. Sete estudos^(23-24,26,28-29,31,33)^ usaram instrumentos qualitativos para avaliar programas de treinamento, mas apenas dois deles^(24,26)^ o fizeram sem uma parte quantitativa. Foram identificadas entrevistas semiestruturadas^(23-24,26)^, questionários com perguntas abertas^(28,33)^, análise descritiva das palavras-chave nos projetos dos alunos^(29)^, pesquisa qualitativa^(31)^, análise qualitativa baseada nas narrativas dos participantes^(29)^ e adesão após o primeiro mês da intervenção^(29)^. O tipo mais predominante de análise qualitativa foi a análise temática^(24,26,28-29)^. A maioria dos estudos não descreveu a confiabilidade ou a validade dos instrumentos qualitativos, com exceção de Koto-Shimada^(24)^, que realizou um teste piloto. Outras medidas relacionadas ao trabalho também foram relatadas, ou seja, registros clínicos e informações de pacientes^(32)^, questionário de exposição autorrelatada a ferimentos por agulhas^(30)^.

A escolha de uma ferramenta de medição é fundamental para avaliar a eficácia de qualquer intervenção, especialmente quando se trata de treinamento. Esta etapa está incorporada no modelo de Kirkpatrick e deve ser cuidadosamente selecionada pelos enfermeiros para qualificar a análise de cada nível. Quanto mais precisa for a ferramenta (ou seja, mais alinhada com o objeto de estudo), maiores serão as chances de uma avaliação confiável. Quanto mais precisa for a ferramenta (ou seja, mais alinhada com o objeto de estudo), maiores serão as chances de uma avaliação confiável.

## Discussão

Esta revisão mostrou que o Kirkpatrick *Framework*tem sido amplamente utilizado na avaliação do treinamento de enfermeiros e também em outras áreas da saúde^(11)^. A amostra retratada neste estudo indicou consistência na conformidade com as diretrizes e os principais resultados do treinamento indicam que este modelo é útil para avaliar a eficácia, com resultados positivos em cada nível, em diferentes tipos de intervenções de treinamento.

O processo de avaliação convergiu com a orientação do modelo que se concentra nos resultados dos participantes^(7)^. Entretanto, um estudo abordou os gerentes dos participantes e surgiu uma visão complementar, conforme exemplificado na citação: “os enfermeiros não sentiram a mudança na atmosfera e nas atitudes da mesma forma que os enfermeiros gerentes haviam observado”^(33)^. A inclusão da perspectiva da liderança pode ser positiva, especialmente para obter *feedback*sobre a transferência de conhecimento para o local de trabalho (nível III) ou mudanças na prática ou resultados organizacionais (nível IV), considerando sua função de supervisão.

O tipo mais comum de informação de linha de base foi no nível de Aprendizagem^(10,23,27,30,32)^. Os níveis de Comportamento e Resultados foram os mais divergentes em relação ao momento. Não há diretrizes no modelo de Kirkpatrick sobre o melhor momento para avaliar cada nível^(7,15)^ e não foi encontrado um momento mais comum ou assertivo para a coleta de dados, já que nenhum dos estudos converge para o melhor momento. Entretanto, é importante ressaltar que os programas de treinamento esperam resultados de curto, médio e longo prazo. Portanto, a seleção de um momento específico (por exemplo, antes, durante, logo após, mais tempo após) ou período (por exemplo, acompanhamentos) é essencial para uma avaliação abrangente que considere que o sentimento de reação diminuirá com o tempo e os resultados mais importantes aparecerão com o tempo^(8)^.

O modelo de Kirkpatrick é uma estrutura flexível e ajustável que incorpora diferentes ferramentas para avaliar os resultados do treinamento (Reação, Aprendizagem, Comportamento e Resultados), o que reforça seu poder, uma vez que as realidades, os treinamentos e os contextos de cada organização são completamente diferentes. Entretanto, justamente por não indicar um padrão metodológico específico, os autores devem ter o cuidado de garantir a validade e a confiabilidade das informações coletadas^(17)^.

Observou-se que tanto o número de estudos quanto a diversidade de instrumentos diminuíram à medida que o nível de avaliação aumentou. A revisão da literatura indica que o nível “Resultados” é o mais difícil de ser alcançado. Reconhece-se que o nível IV “poderia identificar o valor agregado à sociedade de um determinado programa educacional, pois permite avaliar não apenas a aplicação de projetos de intervenção na prática dos profissionais de saúde, mas também os resultados de sua utilização em um contexto local”^(29)^. Entretanto, metade das amostras desta revisão não relatou a medição deste nível. O motivo mais comum relatado para a exclusão do nível IV foi a necessidade de períodos de acompanhamento de médio e longo prazo^(26)^ e a complexidade de relacionar os resultados com o programa de treinamento, distinguindo-os de muitos outros fatores que poderiam afetar os resultados^(31)^. A avaliação de resultados (nível IV) é a mais desafiadora porque deve considerar a transformação ou os impactos do treinamento na prática organizacional, o que exige outros tipos de instrumentos, informações organizacionais e o ponto de vista de outras partes interessadas, além de mais tempo. Nesse sentido, foram encontrados dados semelhantes com relação à relevância percebida dos níveis III e IV e à dificuldade de implementar estes níveis^(17)^.

Há algumas críticas sobre a rigidez deste modelo, que pode fazer com que aspectos essenciais da avaliação, como a visão formativa, não sejam considerados^(16,18)^. Apenas um estudo relatou a coleta de dados em cada módulo e no final do curso no nível das reações^(29)^. Nenhuma outra experiência foi identificada no acompanhamento. Estas evidências mostram que, apesar da diversidade na perspectiva das ferramentas de avaliação de resultados, especialmente com o uso de abordagens qualitativas para captar a perspectiva dos alunos, todas as experiências relatadas foram em uma visão somativa ou transversal.

Os instrutores não estão usando apenas os níveis mais altos, o que é uma preocupação das críticas ao modelo de Kirkpatrick^(16-18)^, como foi observado. Seis artigos conseguiram atingir o nível quatro e outros cinco usaram uma combinação dos três primeiros níveis, representando 85% da amostra. Isso indica que os instrutores de enfermagem estão tentando usar todos os quatro níveis do modelo para coletar informações sobre os resultados, apesar do desafio em relação ao nível de resultados. Isso reafirma o compromisso dos pesquisadores com a recomendação de usar um modelo completo para maior precisão^(7-8)^.

A relevância desta revisão reside no fato de que ela sintetiza o reconhecimento da operacionalização do modelo. Uma grande análise bibliométrica constatou que o modelo de Kirkpatrick era um tópico de interesse em alta após os anos 2000, incluindo 20,7% de sua amostra como algum tipo de publicação de revisão da literatura. No entanto, a maioria dos estudos teve como objetivo criticar o modelo ou identificar seus benefícios, e foram encontradas poucas evidências de revisão sobre os tipos e a caracterização das ferramentas de avaliação e sua aplicação em uma área específica, como a enfermagem^(11)^.

Este estudo tem limitações metodológicas. Os vários projetos de pesquisa das publicações incluídas impossibilitaram a produção de evidências por meio de técnicas de metanálise ou metassíntese. Considerando a natureza da revisão integrativa, o objetivo não foi o de avaliar criticamente a qualidade dos estudos e, por este motivo a qualidade dos estudos não foi usada como critério de inclusão.

Incentiva-se a realização de pesquisas futuras para identificar os melhores construtos para avaliar cada nível, para expandir a implementação do nível IV e para promover a discussão científica sobre a orientação metodológica para compor a cadeia de evidências entre os níveis, conforme proposto pelo modelo *New World Kirkpatrick* ^(15)^. Além disso, a revisão da literatura identificou estudos longitudinais limitados com acompanhamentos consecutivos para a coleta de dados, o que é altamente recomendado para a obtenção de evidências de alto nível em relação às vias causais.

## Conclusão

O modelo de quatro níveis de Kirkpatrick é uma escolha adequada em termos de adaptação dos instrumentos de avaliação ao projeto de treinamento. A seleção de um conjunto confiável de instrumentos é fundamental para qualificar a análise de cada nível, sendo que as escalas Likert são a escolha mais comum para os instrutores. Este modelo é prolífico para avaliar o treinamento na área de enfermagem, porém requer uma escolha cuidadosa dos instrumentos para cada nível. Foram identificadas muitas ferramentas de medição qualitativa e quantitativa que podem ser úteis para profissionais e acadêmicos em avaliações e pesquisas futuras.
